# Emerging Triboelectric Nanogenerators for In‐Body Implantation

**DOI:** 10.1002/smtd.202501443

**Published:** 2025-10-21

**Authors:** Xiao Xiao, Xiangchun Meng, Yong Hyun Kwon, Yoojin Park, Sang‐Woo Kim

**Affiliations:** ^1^ Department of Materials Science and Engineering Yonsei University Seoul 03722 Republic of Korea; ^2^ Center for Human‐Oriented Triboelectric Energy Harvesting Yonsei University Seoul 03722 Republic of Korea

**Keywords:** bioadhesive, implantable, on‐demand applications, stimuli‐responsive, triboelectric nanogenerator

## Abstract

Emerging implantable medical devices (IMDs) collect critical health data for individualized treatment strategies, providing noninvasive, real‐time monitoring to efficiently manage diseases. However, their reliance on conventional batteries introduces surgical risks and operational limitations, necessitating the exploration of alternative energy solutions, such as triboelectric nanogenerators (TENGs). As these implantable TENGs develop, they are increasingly used to power medical devices and function as IMDs themselves. Despite improved design flexibility and compatibility with various functional materials, TENGs still face challenges in maintaining stable operation within the fluid environment in‐body. This review provides an in‐depth look at functional implantable TENGs in biomedical settings, highlighting the basic mechanism of TENGs and covering their electricity generation process, commonly used material combinations, and design methods. The review classifies implantable TENGs into four types: stretchable, bioadhesive, on‐demand biodegradable, and stimuli‐responsive, to address challenges after implantation. Additionally, this article discusses current developments and future possibilities, aiming to showcase new research avenues and opportunities by evaluating both existing applications and potential innovations.

## Graphical abstract

This review explores functional implantable triboelectric nanogenerators in biomedical applications, discussing their mechanisms, electricity generation, and design approaches. To solve postimplantation challenges, four types of triboelectric nanogenerators (TENGs) are introduced: stretchable, bioadhesive, on‐demand biodegradable, and multiresponsive TENGs. Additionally, the review examines recent research advancements and strategic innovations, highlighting current developments and prospects to underscore emerging research opportunities and potential applications.

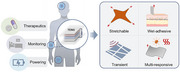

## 1 Introduction

Emerging implantable medical devices (IMDs) are transforming traditional patient health management into a more personalized, digitized, and precise treatment.^[^ ^1^, ^2^, ^3^ ^]^ For instance, in the United States, over 20 million individuals benefit from various IMDs, such as cardiac pacemakers, deep brain neurostimulators, and cochlear implants.^[^ ^4^, ^5^ ^]^ These devices significantly enhance patients' quality of life by precisely monitoring and regulating physiological responses.^[^ ^3^, ^5^ ^]^ Furthermore, new wearable and implantable devices excel at accurately collecting and quantifying vital physiological indicators, such as heart rate,^[^ ^6^ ^]^ pulse pressure,^[^ ^7^, ^8^ ^]^ and respiratory rate,^[^ ^9^, ^10^ ^]^ improving diagnostic accuracy and allowing for the development of personalized treatment plans. These technologies offer remote, noninvasive real‐time monitoring and intervention, making it easier to diagnose early‐stage diseases, manage chronic disorders, and respond quickly to acute health crises, all while satisfying the needs of customized care.^[^ ^11^, ^12^, ^13^, ^14^ ^]^

Despite their significant functional advantages, IMDs rely on conventional batteries that require regular replacement, increasing surgical risks and medical costs.^[^ ^15^, ^16^ ^]^ The size and weight of batteries also limit the miniaturization and weight reduction of these devices, potentially impacting patients' daily activities and comfort.^[^ ^12^, ^15^ ^]^ Moreover, monitoring and treatment devices need a stable and reliable power supply, typically ranging from 1 to 100 µW.^[^ ^16^ ^]^ A primary strategy to address this challenge includes enhancing power capacity and directly harvesting energy from the body or the environment, such as biomechanical energy released through heartbeats, respiration, blood flow, and glucose oxidation‐reduction reactions.^[^ ^1^, ^17^, ^18^, ^19^, ^20^, ^21^, ^22^ ^]^

Several battery‐free approaches have been investigated, each with unique strengths and limitations. Piezoelectric nanogenerators (PENGs) provide stable outputs under repetitive deformations and are particularly suited for cyclic motions, such as heartbeat or respiration.^[^ ^23^, ^24^ ^]^ Thermoelectric generators (TEGs) can deliver continuous power given a sufficient temperature gradient, but the small differences between skin and ambient air or between internal tissues often limit their effectiveness.^[^ ^25^, ^26^ ^]^ Biofuel cells (BFCs) utilize endogenous glucose and oxygen to generate relatively high current densities with good metabolic integration, although long‐term performance is hindered by fluctuating substrate levels, enzyme degradation, and biofouling.^[^ ^27^, ^28^ ^]^ Electromagnetic harvesters (EMs) are capable of producing comparatively large currents, but their dependence on coils and magnets restricts miniaturization and raises concerns over magnetic interference and mechanical reliability during long‐term implantation.^[^ ^29^, ^30^ ^]^ Photovoltaic devices (PVs) exhibit high energy conversion efficiency under illumination, but tissue attenuation, limited optical windows, and safety constraints on light power reduce their applicability for fully implanted systems.^[^ ^31^ ^]^ While all of these modalities show clear promise, their widespread adoption in implantable medical devices remains constrained by frequency dependence, the need for stable gradients or substrates, reliance on external energy inputs, and device dimensions incompatible with minimally invasive implantation.^[^ ^32^, ^33^, ^34^ ^]^

Since 2012, the triboelectric nanogenerator (TENG) has demonstrated its potential as an energy solution.^[^ ^34^ ^]^ Based on the principles of contact electrification and electrostatic coupling, TENGs capture mechanical energy from ambient mechanical activities and convert it into electrical energy, providing a sustainable power supply for IMDs.^[^ ^16^, ^17^, ^35^ ^]^ Additionally, TENGs can harvest typically low‐frequency mechanical energy and convert it into usable electrical energy, utilizing energy generated by human activities or organ movements, as well as external sources, such as ultrasound.^[^ ^36^, ^37^, ^38^, ^39^ ^]^ This broad capability for energy harvesting makes TENGs ideal for integration into personalized medical systems, offering new possibilities as an energy solution for IMDs.

As implantable TENGs progress, they are increasingly being used to power IMDs as well as directly monitor human movement and physiological signs.^[^ ^14^, ^40^, ^41^, ^42^ ^]^ Compared to other IMDs, TENGs offer greater flexibility and multifunctionality in terms of shape, size, and integration.^[^ ^34^, ^43^ ^]^ TENGs are compatible with a wide selection of materials, including organic and inorganic substances, polymers, and hydrogels.^[^ ^44^, ^45^, ^46^, ^47^ ^]^ However, the human body is a complicated environment with body fluids, making it difficult to sustain TENG conformal wet adhesion after implantation.^[^ ^48^, ^49^, ^50^ ^]^ Due to poor wet adhesion, the device struggles to maintain a steady and specified position within the body, potentially compromising its functioning and efficiency. Furthermore, to avoid tissue damage and inflammation from prolonged use, implants need to possess qualities similar to neighboring tissues, particularly softness and stretchability, to be compatible with human tissue.^[^ ^43^, ^51^, ^52^, ^53^ ^]^ Mechanical mismatch is also a serious concern. Most contemporary TENGs are unable to maintain consistent contact separation between friction layers during operation, limiting their sensitivity and responsiveness within the body.^[^ ^53^, ^54^, ^55^ ^]^ Additionally, biocompatibility and biodegradability are critical criteria to examine.^[^ ^56^, ^57^ ^]^ To address these challenges, a variety of functional materials have been incorporated into implantable TENGs to enhance their multifunctionality and applicability in vivo. For instance, bioadhesive TENGs are used to secure devices on wet surfaces, stretchable TENGs enhance the sensitivity of implanted devices, and transient TENGs feature on‐demand biodegradability. These materials not only enhance the performance of TENGs but also expand their potential applications across different fields, including electrotherapy and precision drug delivery. By utilizing these functional materials, TENGs can be precisely engineered to meet specific biomedical needs, offering adaptive solutions that are tailored to the dynamic and complex conditions of the human body.

**Figure 1:**
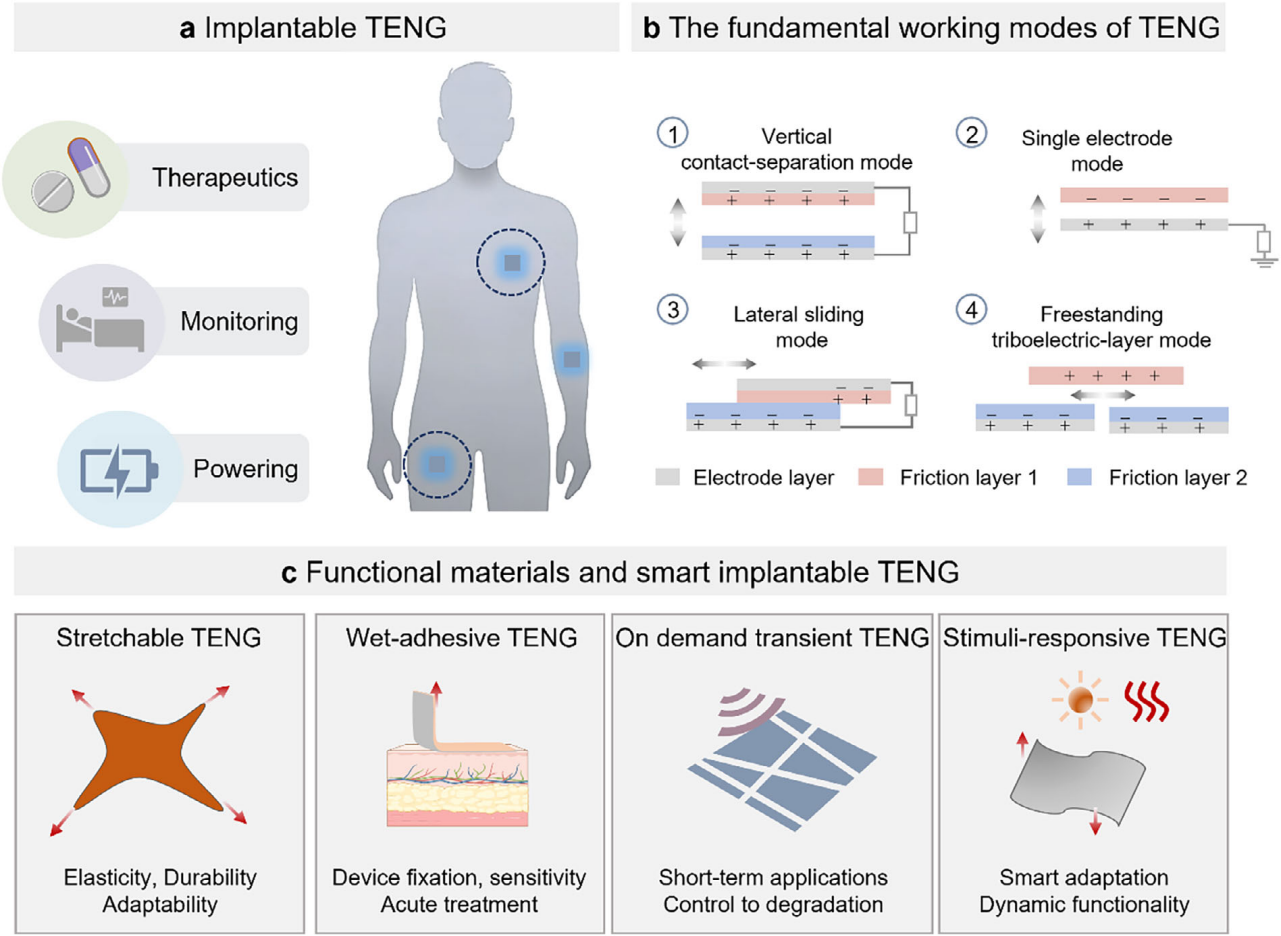
Overall concept of implantable TENGs for in‐body environmental adaptation. a) Implantable TENGs in biomedical applications, featuring capabilities for powering, monitoring, and therapy. b) The four fundamental working modes of TENGs: vertical contact‐separation mode, single electrode mode, lateral sliding mode, and freestanding triboelectric‐layer mode. c) Functional materials and smart implantable TENGs with stretchable, adhesive, on‐demand transient, and stimuli‐responsive materials.

This review provides a comprehensive overview of functional implantable TENGs in biomedical applications, highlighting recent advances in this dynamic domain (**Figure** **1**a). To provide a full introduction to the underlying science and applications, the review delves into TENGs' electricity generation methods, commonly used material pairs, and design methodologies (Figure 1b). On this foundation, functional implantable TENGs are grouped into four categories: stretchable, bioadhesive, biodegradable, and stimuli‐responsive, each designed to address specific requirements after implantation (Figure 1c). Stretchable designs improve mechanical compliance and sensitivity, bioadhesive systems allow reliable fixation on wet tissues, biodegradable devices offer on‐demand resorption to reduce surgical procedures, and stimuli‐responsive constructs adapt to dynamic physiological conditions for multifunctional operation. Together, these developments illustrate how advances in material design and system‐level integration can support the transition of TENGs from experimental studies toward clinically relevant, battery‐free implantable platforms.

## 2 Materials Design Strategy for Implantable TENGs

### 2.1 The Four Fundamental Modes of TENGs

**Figure 2:**
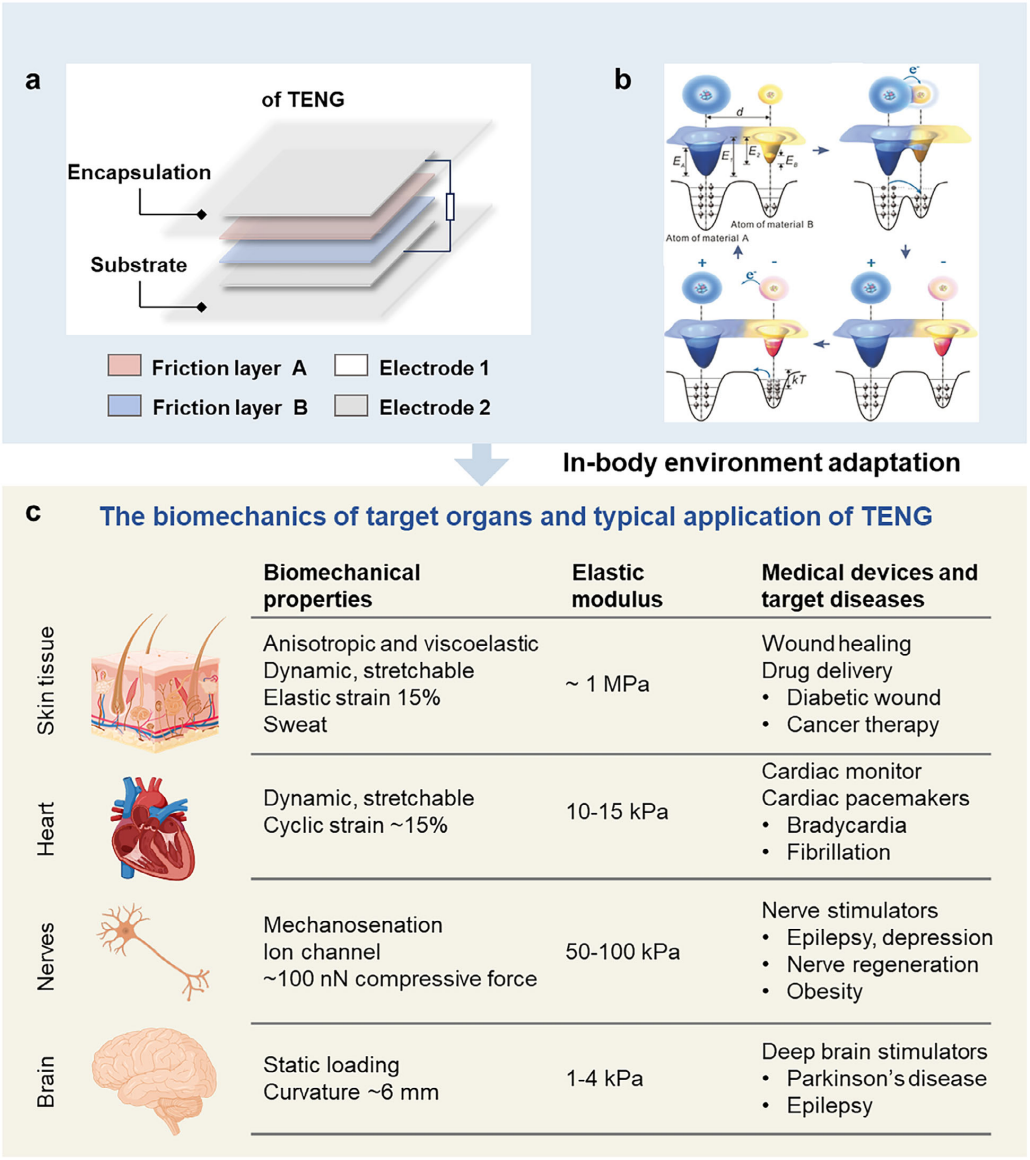
Working mechanism and requirements of implantable TENGs for in‐body environment adaptation. a) The typical device structure of contact‐separation mode TENG. b) Schematic of contact electrification with the atomic origin of contact‐electrification and the overlapped electron cloud model. Reproduced with permission.^[^ ^65^ ^]^ Copyright 2018, Wiley‐VCH. c) The biomechanic properties of target organ and typical applications of TENGs.

As illustrated in Figures 1b and **2**a, the TENG design employs multiple operational modes, each with a unique structure and function tailored to different application requirements.^[^ ^11^ ^]^ The vertical contact‐separation mode generates charge through the contact and separation of two materials under external force. This mode is simple to manufacture, easy to encapsulate, and suited for scenarios requiring rapid response. The lateral sliding mode relies on the horizontal sliding of material surfaces to produce charge, particularly fitting for applications involving continuous actions like vibration or cyclic movement. The single‐electrode mode employs a single moving friction material against a grounded electrode layer, ideal for space‐constrained situations or where device simplification is necessary. The freestanding triboelectric layer mode includes an independent triboelectric layer and multiple electrode layers in a coplanar configuration, with a higher charge transfer efficiency due to the absence of electrostatic shielding effects. Each mode demonstrates the efficiency and flexibility of TENGs as an energy conversion solution, indicating their vast potential across various scenarios.

Recent studies have expanded triboelectric research beyond the four classical modes by introducing new interfacial mechanisms and material systems. Tribovoltaic nanogenerators (TVNGs) use semiconductor contacts where friction excites carriers and built‐in junction fields extract them as direct current; MoS_2_–metal pairs show much higher current density and lower impedance than conventional TENGs, but the open‐circuit voltage is capped by the junction potential and sliding wear/charge trapping limits durability.^[^ ^58^, ^59^ ^]^ Tribo‐photovoltaic systems couple tribo‐induced fields with light excitation: tribo‐induced fields aid photocarrier separation while illumination raises carrier density; heterojunctions and perovskite devices yield higher conversion and simultaneous solar–mechanical harvesting, at the cost of heterostructure complexity and photoactive‐layer stability requirements.^[^ ^60^ ^]^ Solid–liquid TENGs rely on charge transfer in the electrical double layer at solid–liquid interfaces; self‐refreshing surfaces reduce mechanical wear and enable repeatable droplet pulses, although output is highly sensitive to ionic strength/pH and long‐term operation is challenged by electrode corrosion and biofouling.^[^ ^61^, ^62^ ^]^ Hybrid harvesters integrate triboelectric transduction with piezoelectric, electromagnetic, or biofuel modules to widen the usable frequency band and smooth power via rectification/storage, but add circuit overhead and footprint. As a side avenue, tribocatalysis uses tribo‐generated charges/fields on catalytic oxides to drive interfacial redox and form ·OH/·O_2_^−^ for pollutant degradation or H_2_ evolution beneficial chemically, though not a direct contributor to electrical output.^[^ ^63^, ^64^ ^]^ Overall, the field is shifting from single‐effect induction to multifunctional platforms; success in vivo will hinge on interfacial durability, charge retention in wet media, hermetic yet soft encapsulation, and biocompatible/biodegradable materials and by‐products.

### 2.2 Structure and Mechanism of th TENGs

TENGs efficiently convert mechanical energy into electrical energy through the interplay of triboelectric effects and electrostatic induction.^[^ ^34^, ^43^ ^]^ As common in contact‐separation TENGs depicted in Figure 2b, they typically consist of a pair of friction layers, electrodes, an encapsulation layer, and a substrate, producing electrical output upon applied and released deformation.^[^ ^65^ ^]^ During this conversion process, the contact and separation of different materials induce charges, explained theoretically through concepts, such as the Fermi level and surface state models. In these models, localized electrons in specific atoms or molecules form an electron cloud, creating potential wells on the surfaces of materials A and B. When the two materials come into contact, electrons move from potential well A to B, forming an asymmetric double‐well potential, facilitating electron transfer. As the materials separate, the energy barrier of material B retains most of the transferred electrons, leaving material A positively charged and B negatively charged. With an increase in temperature, electron activity intensifies, allowing for easier escape from the potential wells, potentially returning to the original material or releasing into the air, thereby explaining how substances retain the electrons generated by contact electrification.

### 2.3 Requirements of Materials for Implantable TENGs

Implantable TENGs must function reliably in complex physiological environments, which impose two foundational requirements: biocompatibility and mechanical compliance. Biocompatibility ensures that materials and their degradation products remain nontoxic and nonimmunogenic. Even nominally inert substances can trigger protein adsorption, immune activation, and fibrotic encapsulation; thus, careful control of surface chemistry and topography is required to minimize adverse host responses.^[^ ^66^, ^67^ ^]^ Mechanical compliance is equally critical, since soft tissues, such as neural or cortical regions, have moduli in the low‐kPa range, while metals and silicon are orders of magnitude stiffer, leading to pronounced mismatch. Adhesive systems must therefore approximate tissue modulus to mitigate stress concentration and prevent damage.^[^ ^66^ ^]^ Without such compliance, tissue micromotion induces chronic injury, including microglial activation and glial scarring in the brain.^[^ ^66^, ^68^ ^]^ Therefore, TENGs must be specifically designed to adapt to the complex internal environment and meet these demands, thereby enhancing the efficacy of medical devices (Figure 2c).

The skin is anisotropic and viscoelastic, capable of up to 15% elastic stretch with an elastic modulus of about 1 MPa. Applications of TENGs in the skin primarily include promoting wound healing and drug delivery. Cardiac muscle tissue, dynamic and stretchable, has about 15% cyclic strain and an elastic modulus of 10–15 kPa, with TENGs used here for monitoring cardiac activity and as pacemakers. Nerve tissue, requiring a low compressive force around 100 nN and having an elastic modulus between 50 and 100 kPa, uses TENGs for nerve stimulation. Brain tissue, which endures static loading, has an elastic modulus of 1–4 kPa and uses TENGs as deep brain stimulators.^[^ ^1^, ^12^, ^15^, ^21^, ^22^, ^50^, ^52^ ^]^ **Table** **1** below displays various biomedical applications of implantable TENGs and the materials they utilize. However, the stability and functionality of TENGs face challenges within the human body, particularly due to poor wet adhesion caused by the bodily fluid environment, affecting the device's stable position and efficiency. To prevent tissue damage and inflammation from long‐term use, implants need to possess qualities similar to surrounding tissues, particularly in softness and elasticity. Additionally, mechanical mismatches limit the sensitivity and responsiveness of TENGs inside the body. Integrating more functional materials into TENGs is key to solving these issues and significantly enhances their multifunctionality and potential for in‐body applications.

**Table 1:** Biomedical applications of implantable TENGs.

Key materials	Working mode	Output performance	Biocompatible	Energy source	Bioapplication	Refs.
Red blood cells, Reed film, polylactic acid	Contact‐seperation	*V*_oc_ = 0.176 V *I*_sc_ = 0.192 µA	Biodegradable PLA (30 d) Mg (1–3 d)	Body movement	Drug delivery for cancer treatment	[69]
Surgical glove, triboelectric sensors	Contact electrification	100% specificity 98%–100% sensitivity	Biocompatible	Anal sphincter movement	Detect sphincter injuries	[70]
P(VDF‐TrFE), CCTO	Contact electrification	*V*_average_ ≈21.99 V *I*_pp_ = 697 µA	Biocompatible	Ultrasound	Restoring bladder functions	[71]
2‐Hydroxyethyl Methacrylate, PTFE, Au/Cu,	Single‐electrode	*V*_pp_ = 15.5 V *I*_pp_ = 489 µA	Biocompatible	Ultrasound	Charging capacitors	[72]
Passive and counter electrodes	Body‐mediated energy transfer	Loss potential input:20 V	Biocompatible	Body movement	Cell proliferation and differentiation	[73]
PI, Copper electrode	Contact‐separation	*V* = 1.2 V *I* = 150 µA	Biocompatible	Ultrasound	Ultrasound energy harvesting and passive wireless sensing	[74]
FEP, Cu, Al, PDMS	Single‐electrode	1.8 V (DC output)	Biocompatible	Ultrasound	Charging capacitor	[75]
PET, PTFE, PDMS, ITO, biographene	Contact‐separation	*V*_oc_ = 282 V *I*_sc_ = 16.2 µA	Biocompatible	Biomechanical energy from breathing	Charging energy storage device	[76]
PLGA, Mg	Contact‐separation	*V*_pp_ = 4.5 V	Biodegradable	Body motions	Biofeedback bone fracture healing	[77]
Silk aerogel, PTFE filter	Contact‐separation	*V*_oc_ = 52.8 V *I*_sc_ = 5.2 µA	Biocompatible	NA	Charging capacitors	[78]
PTFE, titanium, PDMS, Teflon, Au	Contact‐separation	*V*_oc_ = 65.2 V	Biocompatible	Biomechanical energy	Powering	[29]
Nanoneedle‐array, Cu, PTFE, Acrylic	Freestanding and contact‐separation mode	*V*_oc_ = 20 V I_sc_ = 4 µA	Biocompatible	Biomechanical energy	Drug delivery	[79]
Nanowires, Kapton, Ti	Piezo‐enzymatic ‐ reaction coupling effect	working range: 0.024–0.119 g L^−1^	Biocompatible	Body mechanical energy	Monitor the blood glucose concentration	[80]
PZT, PET, Au	Piezoelectric sensors	Sensitivity: 0.018 kPa^−1^	Biocompatible	Arterial pulse and respiration rat	Arterial pulse monitoring	[81]

## 3 Design Strategy for Functional Implantable TENGs

Flexible IMDs are increasingly pivotal in health monitoring, human–machine interactions, and personal health data management.^[^ ^1^, ^82^, ^83^, ^84^, ^85^ ^]^ TENGs have been successfully integrated into wearable technologies for energy harvesting, robotic control, and various monitoring applications.^[^ ^86^, ^87^, ^88^, ^89^ ^]^ Nevertheless, implantable TENGs face significant challenges, including mechanical mismatches and poor adhesion postimplantation, which adversely affect their conformability and potential internal applications.^[^ ^43^, ^53^, ^77^, ^90^ ^]^ By incorporating specialized functional materials, TENGs can achieve more personalized and precise control.^[^ ^91^, ^92^, ^93^, ^94^ ^]^ This review categorizes implantable TENGs into four distinct types based on their material composition: stretchable TENGs utilizing elastic materials, bioadhesive TENGs employing biocompatible adhesives, on‐demand biodegradable TENGs, and stimuli‐responsive TENGs that interact with stimuli‐responsive materials. Consequently, the enhancement of implantable TENGs with these functional materials leads to intelligent features in biomedical applications, underscoring their expanded capabilities and applications in medical electronics.

### 3.1 Stretchable Implantable TENGs

#### 3.1.1 Stretchable Materials for Implantable TENGs

**Figure 3:**
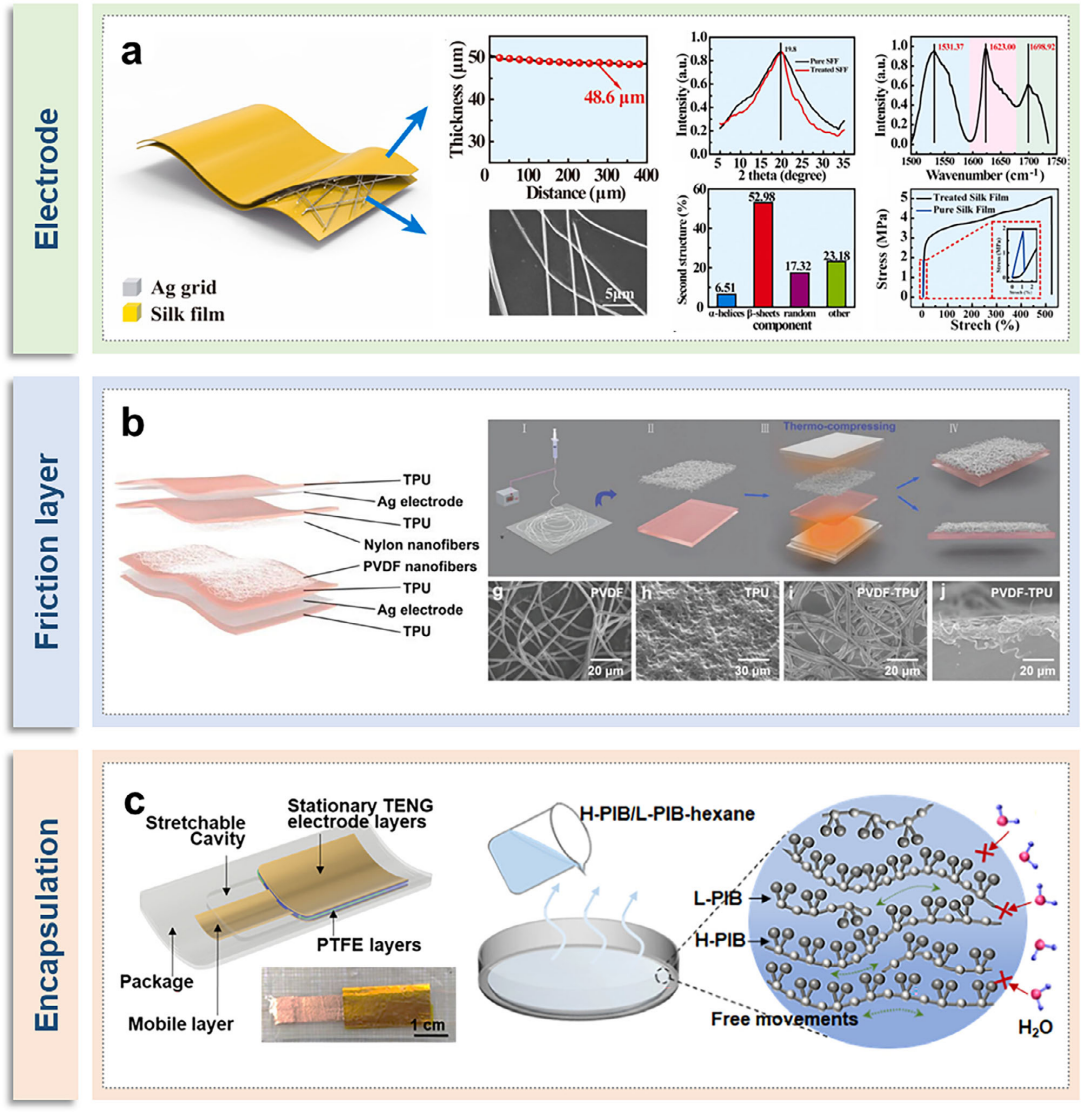
Stretchable materials for implantable TENGs. a) Silk film/Ag grid composites used as the electrode layer of implantable TENGs. Reproduced with permission.^[^ ^95^ ^]^ Copyright 2020, Elsevier. b) PVDF‐TPU film used as the friction layer of implantable TENGs. Reproduced with permission.^[^ ^96^ ^]^ Copyright 2023, Wiley‐VCH.c) Polyisobutylene film used as the encapsulation layer of implantable TENGs. Reproduced with permission.^[^ ^97^ ^]^ Copyright 2022, American Chemical Society.

Typical TENGs are composed of several key components: an encapsulation layer, friction layers, and electrodes. In this review, we have specifically selected stretchable materials for each distinct part of the TENGs. Gong et al. have overcome the issues of poor mechanical flexibility and low electrical conductivity in proteins by developing a high‐transparency, biocompatible, fully degradable, and flexible electrode material for TENGs (**Figure** **3**a).^[^ ^95^ ^]^ The use of stretchable materials is due to the need for electronic skin to possess excellent flexibility, allowing it to conform to the contoured surfaces of human skin, thus enabling the harvesting of biomechanical energy and wireless sensing. This novel protein‐based electronic skin in biomechanical energy harvesting and wireless sensing is primarily reflected in the following aspects.

Chen et al. employed poly(vinylidene fluoride) (PVDF) films and thermoplastic PU (TPU) substrates as friction layers in thermo‐compression (TC)‐TENGs, as depicted in Figure 3b.^[^ ^96^ ^]^ The advantage of these two materials lies in their combined composite film, which exhibits outstanding tensile properties with strains reaching up to 815%. The PVDF film, when tightly bonded with the elastic TPU substrate, provides the composite film with excellent stretchability, and facilitates effective vertical transfer between the two, significantly enlarging the effective area for triboelectric charge generation. Consequently, TENGs based on TC composite films demonstrate a higher output performance (2–4 times) than those of nonlaminated films.

Shao et al. introduced a stretchable encapsulation material with high dynamic waterproofing and tissue‐matching elasticity, providing stability and protection for flexible IMDs, as illustrated in Figure 3c.^[^ ^97^ ^]^ This novel encapsulation material employs a polyisobutylene (PIB) mixture as the elastomer, forming an interwoven network with high‐molecular‐weight H‐PIB to impart the desired extensibility and elastic properties, while low‐molecular‐weight L‐PIB serves as a plasticizer to enhance chain mobility and reduce the elastic modulus. The PIB mixture showcased a superior elastic modulus of 62 kPa in tests, compatible with biological soft tissues, facilitating mechanical performance akin to tissues in medical implants; it demonstrated extremely low water permeability, with rates from 1.6 to 2.9 g m^−^^2^ day^−1^ under stretching states up to 50%, exhibiting excellent dynamic water resistance; the PIB‐encapsulated TENG operated stably underwater for 2 weeks, significantly outperforming the protection provided by other materials. Therefore, the PIB mixture, as an encapsulation material for flexible IMDs, can offer consistent performance to meet the demands of strained physiological environments.

#### 3.1.2 Stretchable Implantable TENG in Biomedical Applications

There is a consistent and growing demand for IMDs for sensing, medical, and energy storage applications that operate independently of batteries.^[^ ^1^ ^]^ These devices are developed for rapid diagnosis and continuous monitoring of critical vital signs. Hence, choosing stretchable materials allows these devices to better conform to the structure of soft tissues, ensuring comfort and adaptability during implantation and use. The scalability and flexibility of these materials allow for seamless integration with skin, tissues, and organs, ensuring wearer comfort during use or implantation and improving the longevity and reliability of the devices.^[^ ^98^, ^99^, ^100^ ^]^ Stretchable TENGs are particularly suitable due to their lightweight structure, simplicity of design, precise capture of low‐frequency movements, rapid responsiveness, and highly sensitive self‐powered sensing capabilities.^[^ ^101^ ^]^

**Figure 4:**
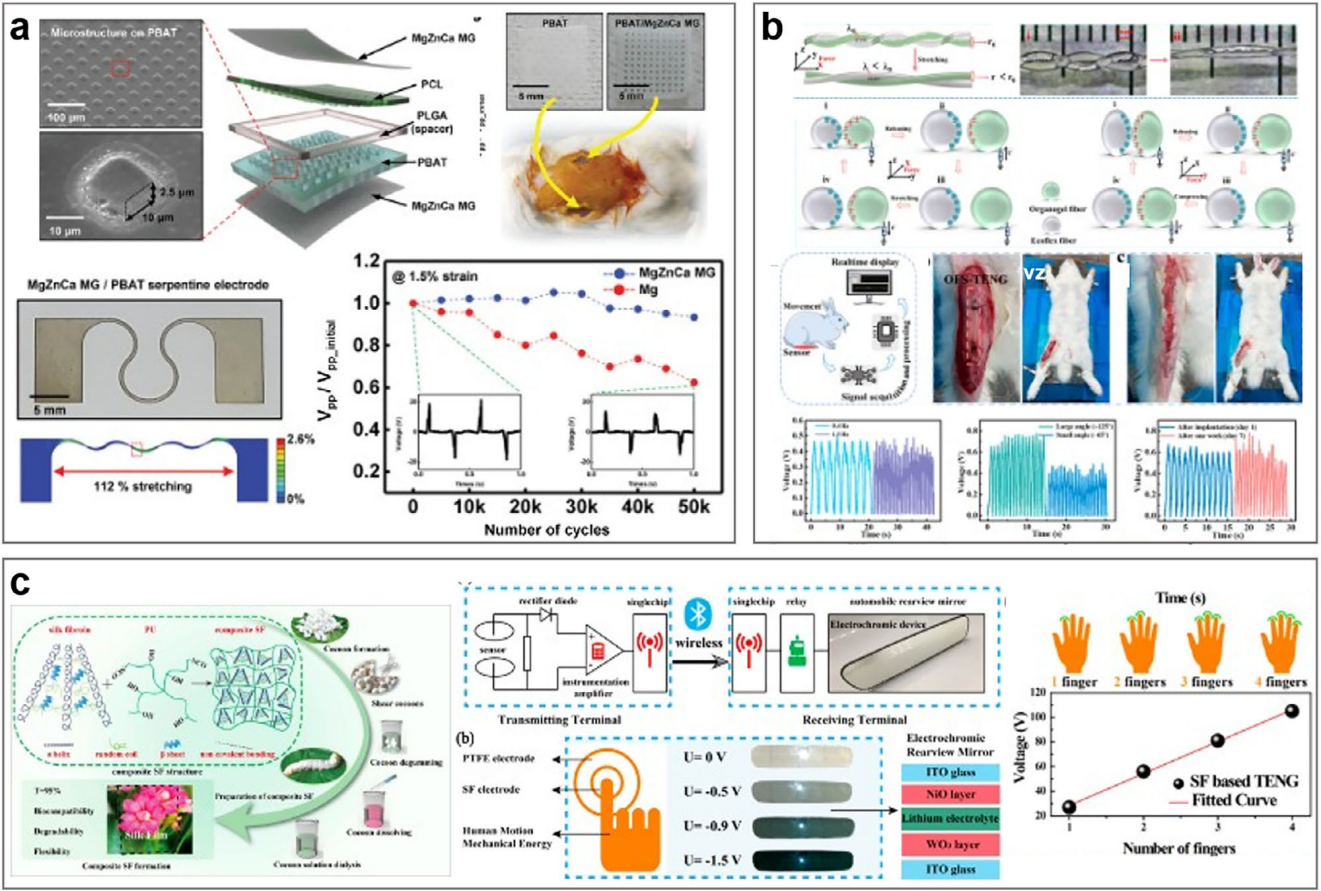
Applications for stretchable implantable TENGs. a) MgZnCa metallic glass enhances stretchable, biocompatible electronics for biosensing. Reproduced with permission.^[^ ^102^ ^]^ Copyright 2021, Wiley‐VCH. b) Ultrastretchable OFS‐TENG sensor monitors ligament strain in real‐time. Reproduced with permission.^[^ ^103^ ^]^ Copyright 2022, American Chemical Society. c) SF‐TENG for biomechanical energy harvesting in wireless communication. Reproduced with permission.^[^ ^104^ ^]^ Copyright 2021, American Chemical Society.

Bae et al. developed a fully biodegradable MgZnCa metallic glass (MG) thin film that capitalizes on the advantages of its amorphous structure without crystalline defects to create highly extensible stretchable electrodes as shown in **Figure** **4**a.^[^ ^102^ ^]^ These electrodes, used in scalable transient electronic devices, exhibit high elastic strain and superior ductility, demonstrating about 2.6% elastic strain and, when combined with serpentine geometric structures, achieve stretchability up to ≈115%. This metallic glass material shows improved fatigue resistance during repeated stretching due to its broad range of elastic strain limits. MgZnCa metallic glass electrodes have been successfully applied in biodegradable electronic components, including capacitors, inductors, diodes, and transistors, facilitating their integration into transient electronic devices. Transient TENGs requiring large, repeatable deformations have validated the stretchability and fatigue resistance of the MgZnCa MG electrodes. Additionally, biocompatibility tests conducted with in vitro cytotoxicity and in vivo inflammation analyses have confirmed the material's biocompatibility, supporting the biocompatible of MgZnCa MG integrated electronic systems. Biodegradable electrostatic nanogenerators made from MgZnCa metallic glass material have shown stable performance in mechanical energy harvesting and demonstrated biocompatibility in biointegration applications.

Sheng et al. introduced an ultrastretchable organic gel/silicone fiber spiral sensor based on a TENG, designed for self‐powered implantable ligament strain monitoring as shown in Figure 4b.^[^ ^103^ ^]^ This sensor demonstrates high stability and ultrastretchability, successfully implanted in rabbit knee ligaments for real‐time monitoring, offering a solution for the diagnosis of muscle and ligament injuries. The stretchable material mentioned in the article consists of intertwined organic gel and silicone fibers, forming a dual‐spiral structure that provides extreme stretchability. This organogel/silicone fiber‐helical sensor (OFS‐TENG) features rapid preparation (15 s), high transparency (over 95%), substantial stretchability (600%), and excellent stability (lasting over 6 months). This self‐powered OFS‐TENG successfully monitors pressure changes on rabbit knee ligaments in real‐time, thus facilitating real‐time monitoring of knee ligament health and providing an effective tool for the real‐time diagnosis of muscle and ligament damage.

To address the inherent brittleness and poor chemical stability of pure silk fibroin films, Xu et al. introduced PU to enhance the performance of regenerated silk fibroin, developing a fully bioabsorbable vertical contact‐separation mode TENG based on doped silk fibroin (SF‐TENG). This TENG is designed for harvesting biomechanical energy for wireless communication, both externally and internally, as depicted in Figure 4c.^[^ ^104^ ^]^ PU interacts with multiple silk fibroin molecules to form a network structure, significantly reinforcing the mechanical properties of the doped silk fibroin film upon polymerization. Experimental results indicate an interaction between PU and silk fibroin. X‐ray diffraction results for PU show a characteristic peak at ≈25 °, suggesting a crystalline region within the PU structure. PU is composed of alternating “soft” segments of polyether and/or polyester with “hard” PU segments. The frequent *─*C═O and *─*NH sites within the hard segments create a rich hydrogen bonding environment, leading to the formation of hard PU crystalline areas. Within the framework of crystallization theory, especially heterogeneous nucleation, these hard crystalline areas of PU can effectively reduce the nucleation barrier of SF molecules through intermolecular hydrogen bonds between the carboxyl and/or amino groups in the silk fibroin molecules and the PU hard crystalline regions. Doping with PU accelerates the crystallization rate of silk fibroin and results in a higher content of β‐crystals in the composite SF‐PU film compared to pure SF films. Meso‐doping of regenerated silk enhances the secondary structural transition, significantly improving the material's mechanical flexibility (stretchable to about 250% and able to withstand 1000 bending cycles) and chemical stability (able to endure environments of 100 °C and pH 3–11). The result is a robust TENG that can be attached to a finger, intelligently controlling the electrochromic function of a rearview mirror to regulate light transmittance by changing the contact force or area. Therefore, this research offers significant potential applications in smart vehicles, smart homes, and healthcare systems.

### 3.2 Bioadhesive Implantable Devices

#### 3.2.1 Bioadhesive Layer for Implantable Devices

During surgeries and device implantations, it is often necessary to establish a robust mechanical interface between damaged tissues or between devices and organs.^[^ ^105^, ^106^, ^107^, ^108^ ^]^ Traditional methods such as suturing and stapling rely on surgical threads and clips. However, these can cause tissue damage and, due to differences in material properties compared to tissues, may lead to severe complications.^[^ ^109^, ^110^ ^]^ In physiological environments, a hydration layer acts as a physical barrier effectively preventing the adhesion of contaminants, such as proteins and cells. This hydration layer provides a protective barrier for various surface‐functionalized biomolecules, such as antimicrobial peptides.^[^ ^110^, ^111^, ^112^ ^]^ Additionally, ion‐rich polymers are widely used to form surfaces that resist foreign body reactions. This hydration layer also necessitates secondary fixation of TENGs after implantation to ensure device stability.

**Figure 5:**
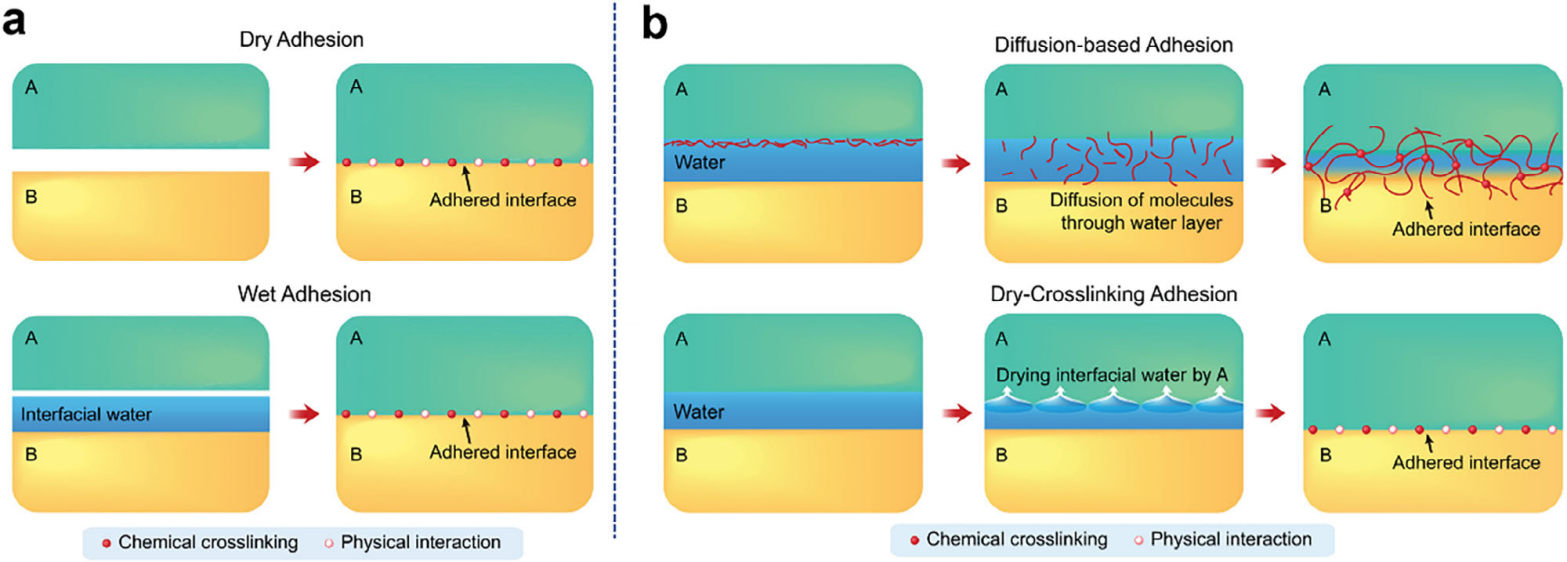
Mechanism of bioadhesion. a) Comparison of dry and wet adhesion mechanisms. b) Mechanisms for wet adhesion: diffusion‐based and dry‐crosslinking. Reproduced with permission.^[^ ^113^ ^]^ Copyright 2020, Elsevier.

As shown in **Figure** **5**a, the common adhesion layers in TENGs are divided into dry adhesion and wet adhesion, where dry adhesion occurs between two dry surfaces and wet adhesion occurs when at least one surface has an interfacial water presence.^[^ ^113^ ^]^ When two dry surfaces come into contact, the functional groups in the adhesive polymer are attracted to the functional groups on the surface of the contact material through mechanisms, such as hydrogen bonding, physical entanglement, electrostatic, and van der Waals interactions, forming immediate adhesion. They can also be physically fixed through mechanical interlocking.^[^ ^114^ ^]^ If interfacial water is present on one or both surfaces, these interactions are significantly hindered because the interfacial water separates the molecules from the two surfaces. The mechanisms of wet adhesion include diffusion and dry crosslinking, as depicted in Figure 5b. In the diffusion mechanism, the adhesive components diffuse across the interfacial water from one adherend, forming physical and/or covalent bonds with the surface or interior of the other adherend through chemical and/or physical interactions. This mechanism can bond wet surfaces together, but due to the low diffusivity of large molecules, diffusion of the adhesive components generally takes a relatively long time. In contrast, in the dry crosslinking mechanism, an adherend quickly absorbs interfacial water, contacts the surface of the other adherend, and subsequently forms stable adhesion through physical interactions and covalent crosslinking, without waiting for the diffusion of adhesive components, thus achieving rapid and strong adhesion of wet surfaces.^[^ ^108^, ^109^, ^112^, ^113^ ^]^

#### 3.2.2 Adhesive Implantable TENG for Biomedical Applications

**Figure 6:**
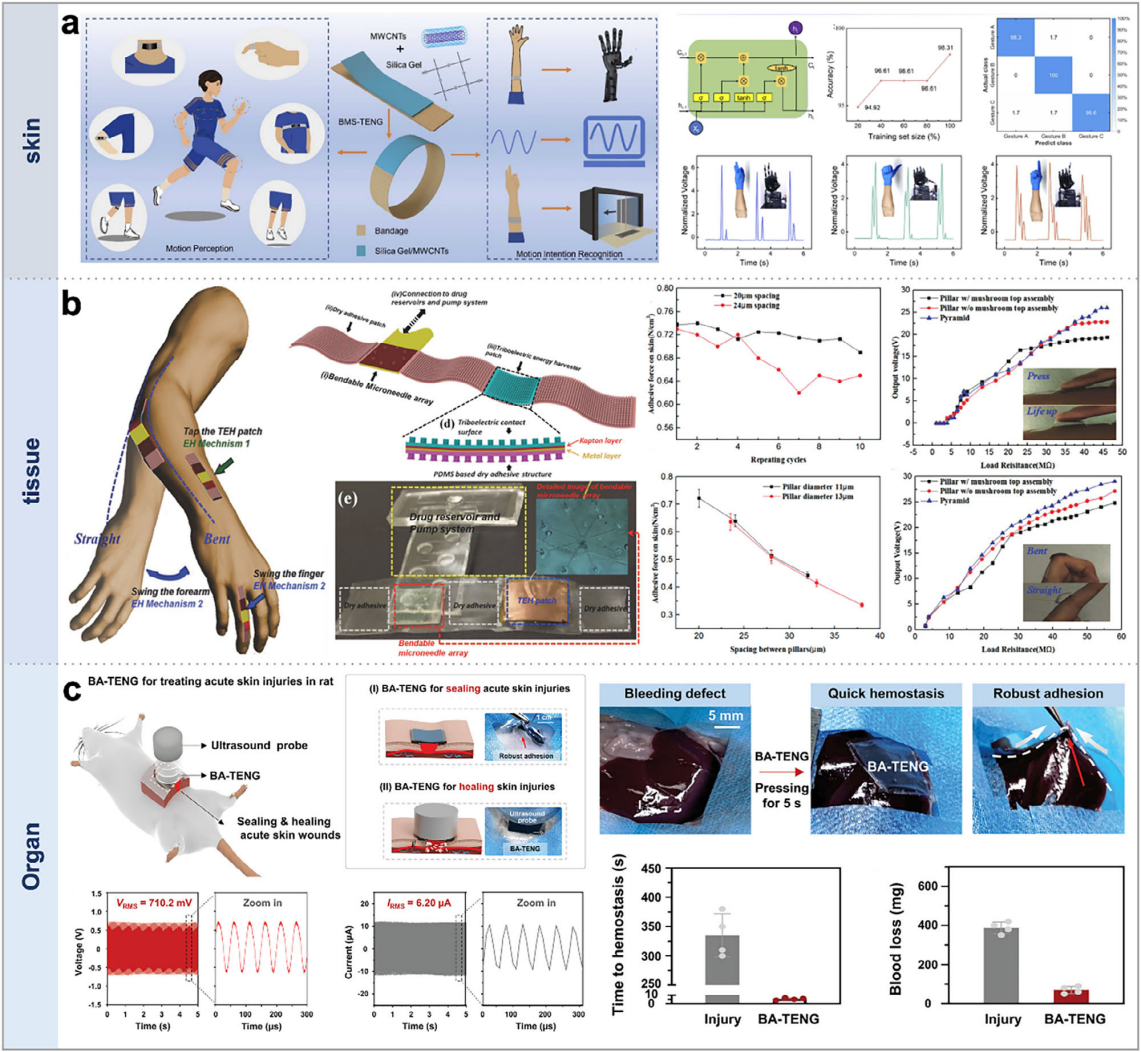
Adhesive TENGs with different implantation sites. a) A flexible smart bandage for motion perception and recognition adhesive on skin. Reproduced with permission.^[^ ^115^ ^]^ Copyright 2023, Elsevier. b) A self‐powered, wearable adhesive patch using a microneedle array for transdermal drug delivery. Reproduced with permission.^[^ ^116^ ^]^ Copyright 2016, Wiley‐VCH. c) A bioadhesive triboelectric nanogenerator for rapid wound closure and accelerated healing. Reproduced with permission.^[^ ^117^ ^]^ Copyright 2023, Wiley‐VCH.

The location of adhesion plays a crucial role in the functionality and effectiveness of adhesive layer selection. Here, we discuss three types of TENGs, each designed to adhere to different biological interfaces: skin, tissue, and organs. Zheng et al. developed a novel flexible, stretchable, self‐adhesive smart bandage, as depicted in **Figure** **6**a.^[^ ^115^ ^]^ This bandage features a self‐adhesive capability that allows it to easily adhere to skin areas requiring movement monitoring, eliminating the need for additional tapes or adhesives, thus reducing skin irritation. The self‐adhesive quality is attributed to the materials and structural design. Specifically, the bandage is crafted from elastic fabric, carbon nanotubes, and silica gel, which not only provide excellent stretchability and air permeability but also ensure a secure fit to the skin. Moreover, the bandage maintains its adhesive properties after multiple applications, enabling continuous monitoring of muscle deformation signals. Overall, the self‐adhesive nature of the sensor minimizes continuous skin irritation and allows for long‐term monitoring of muscle movement signals. This sensor exhibits exceptional elasticity, with a maximum stretchability of 502%. It is utilized for monitoring human motion and physiological signals, such as finger bending, elbow flexion, leg lifting, breathing, and swallowing. Utilizing machine learning techniques, a smart bandage system has been developed that can capture motion signals and recognize movement intentions. By collecting signals near the elbow rather than on the fingers, the smart bandage can identify various gestures and muscle movement characteristics. This system shows immense potential in fields such as human motion monitoring, electronic skin, human–computer interaction, gesture recognition, and biomedicine.

Wang et al. demonstrated a self‐powered wearable adhesive skin patch for transdermal drug delivery, as shown in Figure 6b.^[^ ^116^ ^]^ This patch incorporates a flexible microneedle array, a dry adhesive, and a triboelectric energy harvester (TEH). When adhered to flat skin or joints, it generates electrical power to energize future integrated active components. The basic structure and functionality of the patch are constituted by these elements. The dry adhesive used in the study is inspired by the hierarchical structure of gecko foot hairs. This adhesive offers several advantages over traditional acrylic medical tapes: first, it can be cleaned and reused with restored adhesion after each use; second, its physical structure for generating adhesion is less susceptible to surface contamination, oxidation, and other environmental stimuli; third, the spacers used for ventilation provide enhanced biocompatibility. To ensure the stability of the flexible microneedle array, PDMS (polydimethylsiloxane) pillars were introduced as supports at the base of the microneedles. These pillars can bend moderately when forces exceeding their buckling limit are applied, thus absorbing mechanical strain caused by lateral movements and preventing needle breakage during use.

The research team adjusted the ratio of elastomer to curing agent in the PDMS to control its hardness, finding that a higher concentration of the curing agent significantly increased the hardness of PDMS, which is crucial for successful skin penetration. Furthermore, the study involved mechanical performance testing of microneedles equipped with PDMS bases and SU‐8 tips, documenting changes in bending force and displacement. Analysis of the data from the noncontact, contact, and buckling phases ensured that the microneedles would not bend under forces below a certain threshold. By utilizing PDMS pillars, optimizing the hardness of PDMS, performing strength tests, and selecting appropriate microneedle tips, the stability of the flexible microneedle array was ensured, thus preventing needle breakage, and enhancing the success rate of skin punctures. Therefore, this patch design holds significant potential in drug delivery systems, enabling wearable medication administration. To further enhance its functionality, active components, such as sensors and electronic elements can be integrated to enable real‐time monitoring of drug release and biological parameters, thereby personalizing, and precisely controlling medication delivery. Additionally, integrating microcontrollers and communication modules allows the patch to communicate with smartphones or other devices, supporting remote monitoring, data transfer, and comprehensive health management. This not only provides patients and physicians with more detailed information but also improves the management and adjustment of treatment processes. Considering that energy is key to operating these advanced features, integrating additional energy harvesting modules, such as solar or thermal converters, will further enhance the self‐powering capabilities of the patch, extend its lifespan and facilitate broader application scenarios, thus improving user comfort and convenience.

Meng et al. demonstrated a bioadhesive TENG (BA‐TENG), driven by ultrasound, as illustrated in Figure 6c, designed for immediate wound closure and electrically accelerated healing in emergency scenarios.^[^ ^117^ ^]^ The generator is constructed from biocompatible materials, with a flexible TENG as the top layer and a bioadhesive as the bottom layer, endowing it with strong adhesive capabilities on moist tissues. This design allows for robust, seamless closure, and rapid hemostasis directly on wounds without the need for external devices, effectively reducing blood loss. The ultrasonic stimulation enables the BA‐TENG to generate stable currents and voltages underwater, providing essential electrical support for wound treatment. The role of the adhesive is crucial in this research. Traditional electrical stimulation devices often struggle to attach securely on moist tissues, which can compromise therapeutic effectiveness. In this study, the bioadhesive layer allows the soft and biocompatible TENG device to adhere firmly to moist tissues, enabling rapid hemostasis and wound closure. This adhesive provides key support for fast wound management and electrically accelerated healing. Experimental results demonstrate that BA‐TENG can achieve high interfacial strength (≈150 J m^−2^) on moist tissues within about 5 s, effectively sealing defects. In a liver laceration model in mice, BA‐TENG rapidly closed wounds and reduced bleeding by ≈82%. Overall, BA‐TENG showcases its rapid, convenient, and efficient capabilities in emergency medical and urgent care settings, offering a new reliable solution for managing wounds under urgent conditions. In vivo experiments on mice show that BA‐TENG can immediately seal skin wounds and generate a strong electric field of about 0.86 kV m^−1^ through ultrasound, significantly accelerating the healing process. In vitro studies further confirm that this acceleration is primarily due to the electric field enhancing cell migration and proliferation.

During the early stages of healing, skin cells migrate to the wound area, proliferate rapidly, and differentiate, producing various extracellular matrices and cytokines that promote wound contraction and healing. Moreover, the research indicates that electrical stimulation or electric fields can enhance intercellular communication and material transfer, which are critical for cell proliferation and differentiation behaviors, particularly in tissue engineering and wound healing processes. Thus, the strong electric field produced by ultrasound significantly promotes skin wound healing. Beyond wound treatment, this type of TENG also holds potential for applications in nerve stimulation and regeneration, as well as charging batteries in implanted devices, demonstrating its broad application prospects.

### 3.3 On‐Demand Biodegradable Implantable TENGs

Transient bioabsorbable IMDs are designed to degrade over time and be absorbed in vivo, contrasting with traditional long‐term implants used for short‐term therapeutic applications.^[^ ^118^, ^119^, ^120^ ^]^ The degradation performance of bioabsorbable materials varies due to their unique mechanisms, kinetics, and environmental conditions.^[^ ^121^, ^122^, ^123^ ^]^ The lifespan of these systems is entirely dependent on predefined properties, such as material characteristics, sample size, surface morphology, and environmental factors.^[^ ^123^, ^124^, ^125^ ^]^ Currently, most bioabsorbable implants employ passive degradation methods, typically achieved by selecting or modifying materials to facilitate their application in biomedical fields.

Precise control over the lifespan of bioabsorbable TENGs is critical for ensuring clinical safety and practical application. Unlike traditional devices that degrade gradually within the body, on‐demand transient IMDs can degrade swiftly upon signal reception, ensuring operational functionality and minimizing the risk of internal residue. These devices have a lifecycle comprising two stages: initially, the implant materials maintain their functionality with slow quality loss; subsequently, upon triggering by external stimuli, they rapidly disintegrate and dissolve.^[^ ^125^, ^126^ ^]^ This review introduces various techniques for initiating degradation, including ultrasound‐triggered, near‐infrared (NIR) assisted photothermal‐controlled, light‐triggered, and lipase‐triggered degradation of TENGs.

**Figure 7:**
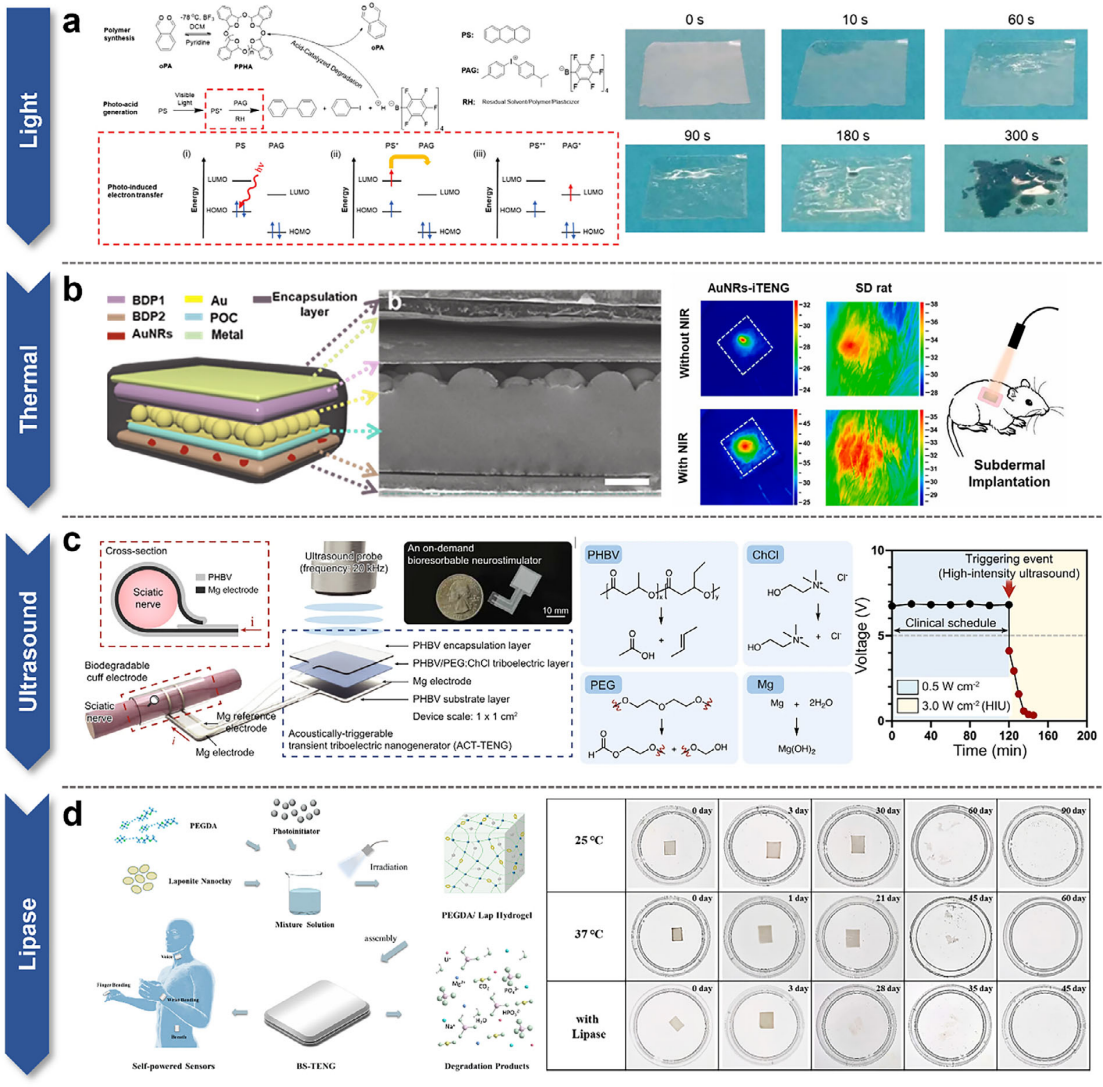
On‐demand Implantable TENGs with various triggering mechanisms. a) A transient TENG using acid‐sensitive PPHA integrated with PAG and PS, degrading under UV light. Reproduced with permission.^[^ ^127^ ^]^ Copyright 2019, Wiley‐VCH. b) Photothermally tunable degradation of a PLA‐based B‐TENG utilizing NIR treatment. Reproduced with permission.^[^ ^128^ ^]^ Copyright 2018, Elsevier. c) Ultrasound‐triggered on‐demand transience of a neural stimulator. Reproduced with permission.^[^ ^129^ ^]^ Copyright 2023, Springer Nature. d) A controllable, biodegradable BS‐TENG designed for self‐powered sensing and electrostimulation therapies, biodegrading via lipase. Reproduced with permission.^[^ ^130^ ^]^ Copyright 2023, American Chemical Society.

Wu et al. developed a transient TENG using acid‐sensitive poly(para‐xylylene) (PPHA) as the substrate (**Figure** **7**a).^[^ ^127^ ^]^ This device also integrates a photoacid generator (PAG) and a photosensitizer (PS). Under UV irradiation, PS absorbs energy, which is then transferred to PAG through a photo‐induced electron transfer reaction, triggering the generation of a photoacid that initiates the depolymerization of PPHA. Additionally, by embedding silver nanowires into the PPHA‐based film, they successfully demonstrated the potential of this transient TENG as a mechanical energy harvester and as a touch/sound sensor. The sunlight‐induced degradation process is facilitated through a photo‐induced electron transfer reaction. In this system, circular PPHA with a low glass transition temperature (‐43 °C) is used as the primary substrate, in conjunction with a photoacid‐sensitive PAG and PS. Here, PS absorbs energy from the UV spectrum (365–400 nm) and transfers it to PAG, which then produces photoacid, catalyzing the degradation of PPHA. Specifically, the photosensitizer, upon absorbing UV light, acts as an electron donor and transfers electrons to the lower energy molecular orbitals of PAG. This thermodynamically favorable redox reaction leads to the dissociation of PAG radicals and the formation of a strong superacid, rapidly degrading the material. This mechanism allows the circular PPHA substrate to degrade quickly under light exposure. By adjusting the ratio of photosensitizer to PAG, the degradation rate can also be precisely controlled, offering finer control over the degradation process. This research not only expands the applicability of TENG as a short‐term power source and sensor but also extends the use of transient functional polymers to more advanced energy and sensing applications.

Li et al. focused on developing a tunable biodegradable implantable TENG (BD‐iTENG) with a hemispherical array structure comprising a poly(1,8‐octanediol‐*co*‐citric acid) (POC) triboelectric layer, electrodes, and gold nanorods (Au‐NRs) as shown in Figure 7b.^[^ ^128^ ^]^ Without NIR treatment, the implanted BD‐iTENG operated normally for over 28 days. However, upon applying NIR treatment, the output voltage of the AuNRs‐BD‐iTENG rapidly dropped to zero within 24 h, and most of the device degraded within 14 days. This rapid degradation is attributed to the Au‐NRs converting NIR into thermal energy, thereby elevating the local temperature of the device. The small radius of Au‐NRs enhances the energy conversion efficiency, generating sufficient heat to trigger the biodegradation of POC. A 5‐min exposure to NIR laser (808 nm, 5.2 W cm^−2^) caused a temperature increase of 13.2 °C, indicating that the degradation of BD‐iTENG could be rapidly initiated and effectively managed in vivo. Moreover, BD‐iTENG generated a maximum output voltage of 28 V in vitro and 2 V in vivo. Additionally, the output performance of BD‐iTENGs significantly enhanced cell migration and wound healing processes. These findings validate the feasibility of using photothermally adjustable BD‐iTENG as a transient power source for biomedical electronic devices.

Lee et al. developed an on‐demand absorbable neural stimulator to address the challenges associated with bioabsorbable bioelectronic devices in medical applications (Figure 7c).^[^ ^129^ ^]^ This neural stimulation system comprises two main components: an acoustically triggerable transient TENG (ACT–TENG) and a bioresorbable cuff electrode. The ACT–TENG, a bioabsorbable energy harvesting system, is activated by ultrasound which facilitates high‐frequency contact and separation between PHBV/PEG:ChCl composite materials and magnesium (Mg) electrodes, generating frictional charges that produce voltage peaks up to 7.8 V and current peaks of 60.0 µA. To assure precision and controllability in clinical applications, as well as quick dissolution, when necessary, hence reducing potential health hazards, the system uses high‐intensity ultrasound (HIU) to speed the mechanical deterioration of the stimulator. HIU generates focused acoustic pressure within the porous structure of the PHBV encapsulation layer, creating favorable conditions for degradation. This pressure‐driven increase in surface area further promotes the hydrolytic dissolution of the material. Experimental results demonstrated that, in deionized water, the neural stimulator rapidly completed mechanical degradation and fully dissolved within 120 min following HIU treatment. Without HIU stimulation, the stimulator maintained structural stability in diluted phosphate‐buffered saline (PBS, pH 7.4) for up to 20 days. Thus, ultrasound stimulation significantly accelerates the degradation and dissolution processes of the neural stimulator. The electrical energy generated by ACT–TENG is transmitted to the target site via the absorbable electrode, achieving precise electrotherapy for peripheral nerve diseases. Additionally, the system's effectiveness in treating compressive peripheral neuropathies and hereditary peripheral nerve disorders has been validated.

Li et al. developed a controllable biodegradable single‐electrode TENG (BS‐TENG) based on PEGDA/Lap hydrogel, which exhibits excellent flexibility, mechanical properties, and electrical performance, suitable for self‐powered sensing and electrostimulation therapies (Figure 7d).^[^ ^130^ ^]^ The key to controlling the degradation process lies in the material used, namely a PEGDA/Laponite nanocomposite hydrogel. By adjusting the temperature or adding lipase, the degradation rate of BS‐TENG can be effectively controlled. Specifically, the degradation of PEGDA/Lap hydrogel undergoes three stages: swelling, cracking, and degradation. During the swelling stage, as the temperature increases, water molecules more rapidly penetrate the hydrogel, quickly reaching swelling equilibrium. The increase in temperature also weakens the secondary interactions between Laponite nanoparticles and PEGDA chains, causing the long PEGDA polymers to break down into shorter chains, thus leading to material cracking. As degradation progresses, the proportion of activated molecules increases with temperature, enhancing the rate of effective molecular collisions and accelerating the chemical reaction rate, resulting in the material dissolving more rapidly in solution. Through careful design and composition, the rate and process of biodegradation can be precisely controlled, ensuring the controllability of the entire degradation process. Moreover, in designing BS‐TENG, this special nanocomposite hydrogel was utilized to ensure sustainable operation and predictable biodegradability in biological environments. The PEGDA/Laponite hydrogel demonstrates exceptional extensibility, toughness, tensile strength, and high electrical conductivity. Notably, when Laponite content is at 10 wt%, the composite hydrogel shows optimal electrical and mechanical properties, with an elongation rate of 1001.8% and a resistance of only 10.8 Ω. The hydrogel material is highly biocompatible with human tissues, effectively promoting cell adhesion. BS‐TENG is also better able to conform to the shapes of human tissues, enhancing its ability to match human tissue contours. This capability plays a significant role in medical diagnostics and therapies.

### 3.4 Stimuli‐Responsive Materials for Implantable TENGs

Stimuli‐responsive materials can detect changes in the environment or external stimuli due to their adaptability, self‐awareness, and memory capabilities.^[^ ^131^, ^132^ ^]^ Combined with TENGs, these materials can respond to changes in external stimuli, such as light,^[^ ^133^ ^]^ temperature,^[^ ^134^ ^]^ humidity,^[^ ^134^, ^135^, ^136^ ^]^ or magnetic fields thus enabling functionalities such as self‐healing, electrochromic, shape memory, photodetection, and thermal response. This technology not only enhances the efficiency of energy collection but also improves the adaptability and functionality of devices. In the realm of implantable devices, such as cardiac pacemakers or neurostimulators, these integrated materials significantly increase energy efficiency, reliability, and patient comfort.^[^ ^138^, ^139^, ^140^, ^141^ ^]^ Additionally, these intelligent materials can sense physiological changes in real‐time and relay this information to external monitoring systems, such as glucose and heart rate monitors, or adjust drug release and mechanical stimulation based on physiological signals, facilitating more personalized and precise treatments.

**Figure 8:**
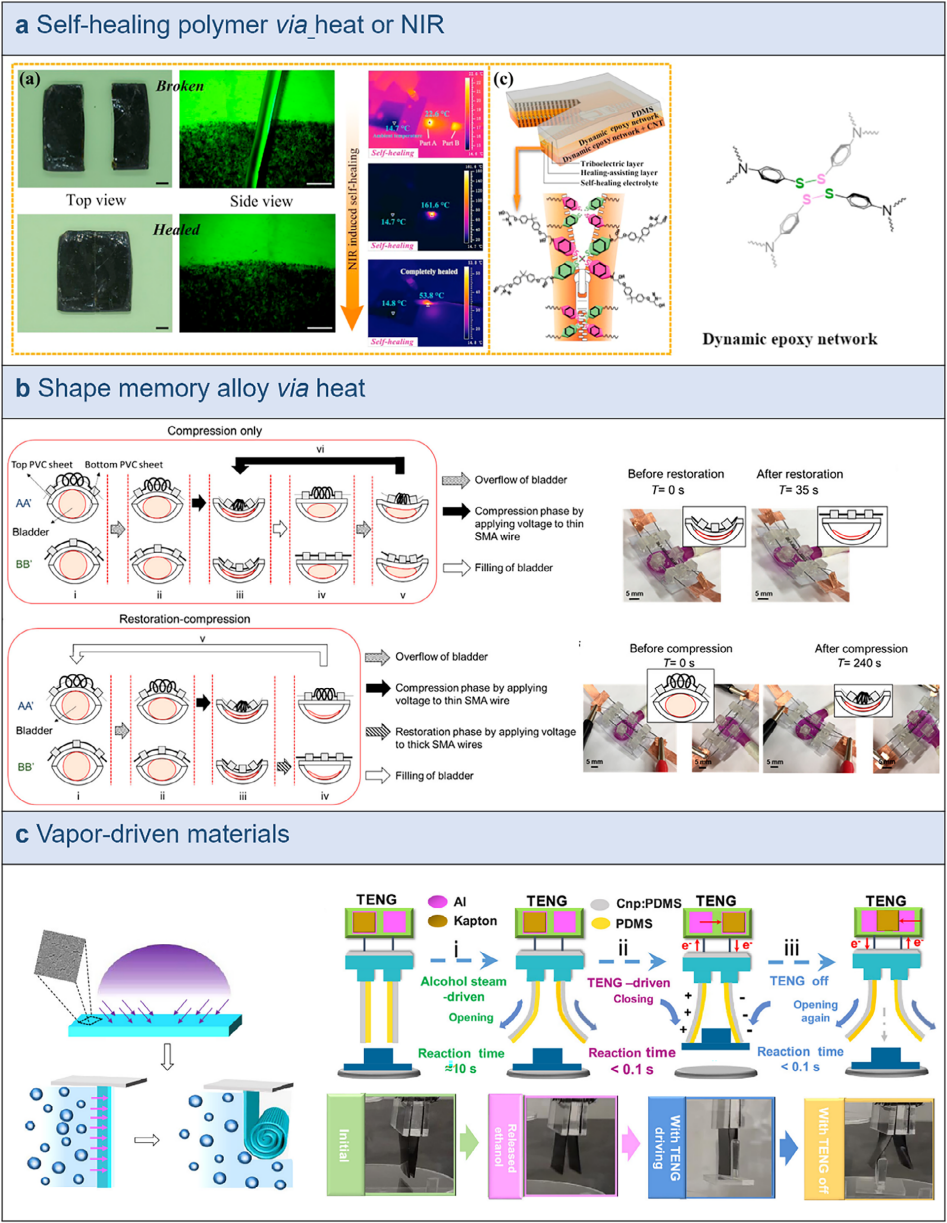
Stimuli‐responsive TENGs. a) Chemical structure and thermal analysis of the self‐healing layers alongside characterization and performance assessment of the self‐healable layer with NIR irradiation. Reproduced with permission.^[^ ^142^ ^]^ Copyright 2018, Elsevier. b) Working mechanism of shape memory alloy NiTi spring actuator and the actuator/balloon before and after restoration or compression with insets. Reproduced with permission.^[^ ^143^ ^]^ Copyright 2018, American Chemical Society. c) Schematic diagram about the curing and deformation processes of the PDMS film, alongside a dual‐stimuli flexible gripper that operates based on TENG and vapor induction. Reproduced with permission.^[^ ^144^ ^]^ Copyright 2019, American Chemical Society.

In **Figure** **8**a, Guan et al. demonstrated a self‐healing triboelectric nanogenerator (SH‐TENG) generated through NIR irradiation by embedding a carbon nanotube (CNT) in a vitrimer elastomer that contains disulfide bonds.^[^ ^142^ ^]^ For IMDs, which function either internally or subcutaneously within the human body and are prone to mechanical wear or injury, the capacity of these materials to heal itself is essential. Devices with self‐healing capabilities have a longer lifespan and require less maintenance since they can promptly rebuild their structure and functionality after being damaged. While intrinsic healing systems comprise components with dynamic covalent or noncovalent connections that assist rearrange the network under diverse stimuli (such as light, pH, or heat), external polymer systems use healing chemicals to restore themselves. Hydrogen bonds, which are weaker and have worse mechanical properties, constitute the basis for many self‐healing materials utilized in implantable devices. These restrictions are overcome by the introduction of dynamic disulfide bonds, which offer strong covalent bonding. Heat may be remotely created inside the SH‐TENG through a dynamic disulfide bond exchange reaction triggered by NIR radiation.

The dynamic disulfide bond reformation method and the multilayer structure design give this TENG exceptional durability as well as quick structural and functional recovery capabilities. This remote, quick, and effective self‐healing procedure has been effectively executed by combining CNTs with vitreous state elastomers that have disulfide connections, indicating its possible use in implantable electronic devices. With NIR stimuli, the SH‐TENG can repair scratches, cracks, and even breaks in a remarkably short amount of time, demonstrating remarkable self‐repair capabilities. Its ability to propagate over long distances makes it possible for it to be remotely controlled and self‐healed even in harsh environments. This characteristic opens new opportunities for implantable devices, allowing systems such as the SH‐TENG to be employed in vivo. Consequently, SH‐TENG offers distinct benefits and a wide range of potential applications in the realm of implantable electronic devices as a self‐healing TENG.

Hassani et al. have developed an innovative system that integrates a TENG with a bistable microactuator, utilizing shape memory alloy (SMA) components to enable autonomous urination on a flexible polyvinyl chloride (PVC) sheet in Figure 8b.^[^ ^143^ ^]^ The core of this system is a highly efficient actuator comprising microdiameter nickel‐titanium (NiTi) shape memory alloy wires and springs, controlled via thermal activation to manage the urination mechanism. The NiTi shape memory alloy spring is stretched and anchored onto the PVC sheet using two 3D‐printed anchor points. The system employs voltage to heat the thin SMA wire inside the NiTi spring, thus shortening the spring. This change drives the physical displacement of the PVC sheet, simulating the bladder compression necessary to facilitate urine expulsion. When the NiTi spring is heated to 45 °C, its length decreases, causing the spring to contract and thus move the PVC sheet, partially emptying the bladder. The design of this actuator considers both energy efficiency and functionality, reducing power consumption during compression and recovery phases, and enhancing contraction force while maintaining the same voltage. Moreover, the system's scalability allows for customization to accommodate larger bladder sizes for different patients. All 3D‐printed components used for fixation and support are made from biocompatible materials to minimize potential irritation to the human body, providing necessary mechanical support and ensuring compatibility with human tissue. Research indicates that this actuator can achieve up to 78% bladder volume emptying within just 20 s of activation, demonstrating efficiency comparable to conventional catheterization methods. Compared to traditional methods that require frequent catheter use, this one‐time implantable device significantly reduces the frequency of catheter use, thereby avoiding associated complications and inconvenience. Furthermore, the integrated TENG and actuator system can accurately monitor bladder fullness, offering the possibility of autonomous urinary control for patients with underactive bladder (UAB). The sensitivity and response speed of this sensor provide a crucial technological foundation for future self‐controlled urination systems, allowing patients to adjust urination timing according to their needs, thereby enhancing quality of life. By adjusting the number and configuration of NiTi spring strips, this device can be easily scaled up to larger animal models or human applications, further improving emptying efficiency and adaptability.

Zheng et al developed a dual‐stimuli intelligent actuator and robotic hand based on vapor‐responsive PDMS films and a TENG (Figure 8c).^[^ ^144^ ^]^ This design integrates vapor‐responsive PDMS films with TENG to create flexible actuators for transporting objects and dual‐finger grippers for handling small items. The study revealed that PDMS films treated with UV/O_3_ undergo spontaneous curling deformation when exposed to small alcohol molecules, such as ethanol vapor. This deformation is controlled by several factors, including the thickness of the PDMS film, the duration of UV exposure, and the concentration of ethanol in the environment. Optimal curling force and response speed were achieved with a film thickness of 1.5 mm and UV exposure of ≈15 min, demonstrating excellent curling performance and fatigue resistance. The ethanol vapor‐induced deformation behavior is utilized to activate intelligent actuator devices. By integrating ethanol vapor‐responsive PDMS with TENG, the researchers designed flexible actuators and grippers that adapt to the size and shape of various objects, with ethanol vapor stimulation aiding in adjusting the configuration of these intelligent actuators, while the electrostatic force provided by TENG enables rapid and controllable actuation movements. Additionally, these devices leverage vapor stimulation and electrostatic force to adapt and manipulate various objects; for instance, the dual‐finger gripper can effectively grasp objects weighing up to 6 g. Apart from TENGs, various multistimuli‐responsive materials are also applicable in diverse implantable energy harvesting systems. A notable example is a mini‐generator that harnesses the body's intrinsic energy to provide a sustainable power source for implantable devices, thus prolonging their operational lifespan and reducing the need for surgical battery replacements.^[^ ^145^ ^]^ This generator operates based on the differential pressure between systolic and diastolic phases. It is equipped with a super hydrophilic surface featuring vertically mobile bubbles that respond to pressure fluctuations, a magnet encased in a superhydrophobic coating, and a system to regulate ambient pressure. Activated by magnetic fields, this device efficiently converts mechanical energy into electrical current.

Stimuli‐responsive implantable TENGs dynamically adapt to disease environments and are increasingly applied in biomedicine. In wound healing, pH‐responsive nanocomposite films (HAP/SN‐NR) driven by TENGs provide continuous electrical cues to enhance cell proliferation, while releasing polydopamine/Fe^3+^ nanoparticles under acidic conditions to clear ROS; strong wet adhesion, self‐healing, and biodegradability further accelerate skin repair and suppress inflammation.^[^ ^146^ ^]^ In trauma care, shape‐memory adhesives seal bleeding wounds rapidly, and ultrasound‐driven TENGs deliver localized electrical stimulation to promote regeneration.^[^ ^147^ ^]^ For drug delivery, magneto‐responsive sponge TENGs (MS‐TENGs) integrate NdFeB particles into porous Ecoflex, enabling reversible compression under magnetic fields to modulate pore volume and drug release; cumulative release reached 65.8 µg in 40 min with magnetic actuation versus 40.3 µg without, while TENG signals allow real‐time monitoring and feedback control.^[^ ^148^ ^]^ In regenerative medicine, a wearable TENG coupled with PLA/Au/PPy microneedles loaded with TRAM1‐EVs enables motion‐driven electrical pulses to switch PPy redox states, weakening EV binding and releasing them through microchannels into NP cells, thereby suppressing inflammatory senescence and repairing intervertebral discs.^[^ ^149^, ^150^ ^]^ These examples show how pH‐, magneto‐, and electro‐responsive materials extend implantable TENGs from power harvesters to adaptive therapeutic systems. As shown in **Table** **2**, a comparative overview of different functional TENGs used in implantable applications is presented, summarizing their representative materials and mechanical properties.

**Table 2:** Comparison of different types of functional TENGs.

Types	Key materials	Output	Feature	Application	Implantation	Refs.
	PHBV/PVA/Mg	*V* ≈4 V (water, 2 W cm^−2^) *I*_sc_ ≈22 µA. Ultrasound‐driven	US‐triggered <120 min for degradation 99%–100% sterilization	SSI prevention	Subcutaneous Pig skin ex vivo US sterilization	[126]
	PHBV/PEG:ChCl/M g	*V*_pp_ ≈7.8 V *I*_pp_ ≈60 µA. Ultrasound (≤1 W cm^−2^);	HIU >3 W‐Cffi'^2^ for degradation	Nerve repair CMT1A	Mice	[129]
On demand biodegradable	PEGDA/Laponite	V_oc_≈10.4–11.2 V *I*_sc_ 1.9 µA Biomechanical	Degradation: Euzyme/temperature, 45‐90d Strain ‐1000%	Wearable implantable biosensors	PBS: 90 d;37 °C:60d; Enzyme: 45 d In vitro only	[130]
	PCL/Mg hCOF nano fibers CZIF‐D	*V*_oc_‐3.92 V (2 W cm^−2^) Ultrasound‐driven	≈35 d for degradation Chemo + electrical synergy	Local tumor therapy	Tumor site, 35 d Mice (C6. 4T1 tumor's) inhibition ‐86%	[151]
	PCL‐r‐PU PAV/Mo	*V*_pp_ ≈1.5 V, *I*_pp_ ≈24.20 µAUltrasound‐driven	Adhesion: 5 s, toughness 150 J m^−2^	Hemostasis wound closure	Rat wound model	[117]
Bio adhesive	B ‐ SMP/PAV/Mo	*V*_pp_ ≈1.2 V (0.5 W cm^2^) Ultrasound‐driven	Adhesion 5 s toughness 150 J‐nr^2^	Wound healing scar reduction	Rat incision 14 d	[147]
	ACMO/PC organogel/LiTFSI/PDMS	Strain‐dependent voltage stable ≥3×10^4^ cycles Ligament/tendon motion	Stretchability: ‐600% Fatigue life: ≥3 × 10^4^ cycles	Tendon ligament monitoring	Rabbit knee ligament	[103]
Stretchable	PDMS/Cu/PI	*V*_pp_ 180 V, *I*_pp_ ≈2.2 µA Body motion + skin friction	Stretchability: ≈30% Durability: ≥3000 cycles	Wearable LED power	Human skin‐mounted	[152]
	PTFE/PFA/PDMS	*V*_oc_ 395–401 V, *I*_sc_ 2.3 µA, Wearable motion	Blue light trigger's NO Stretchability: 2420%	Local tumor therapy	Implanted LED, 21 d Mouse (4T1): inhibition 90.9% Rat glioma: tumor cleared	[153]
	NdFeB/liquid metal	*V*_oc_ 97 V, *I*_sc_ 1.9 µA, Mechanical + magnetic actuation	Magnetic field deformation Drug release	GI drug delivery soft robotics	GI sim: acid 24 h, mass loss 0.9% In vitro drug release	[148]
Stimuli‐responsive	PEDOT: PSS/P VA/M WCNT + Ecoflex	*V*_oc_ 270.5 V. *I*_sc_ 16.2 µA Stretching, bending, pressure	Self‐healing multimodal sensing	Wearable health monitor	Wearable	[154]
	PU/PI/nylon	*V*_oc_ 185 V. *I*_sc_ 36 µA Motion + thermo‐switch	Stimulus: ON <0 °C OFF ≈60 °C Repeatable ON/OFF switching	Smart insole thermal management	Wearable	[155]

## 4 Conclusion and Perspectives

Implantable TENGs have drawn increasing attention as self‐powered platforms in medical electronics. This review has outlined their principles, material strategies, and biomedical applications, with particular focus on functional materials, such as stretchable, bio‐adhesive, degradable, and stimuli‐responsive systems. These approaches improve adaptability and stability in vivo, making TENGs more suitable for powering implants, as well as for health monitoring, therapy, and diagnostics. Nevertheless, several barriers remain before clinical translation can be realized.

### 4.1 New Materials Development

Functional materials have improved biocompatibility and implantation stability, but their long‐term reliability under physiological conditions is not well established. Friction layers and electrodes degrade under cyclic stress and exposure to fluids, and polymers often swell or lose triboelectric performance.^[^ ^3^, ^67^, ^125^ ^]^ Although bioresorbable systems avoid retrieval surgery, predictable degradation kinetics and fully biocompatible byproducts are still unresolved. Future work should focus on durable interfaces with resistance to wear and corrosion, degradable systems whose kinetics are quantitatively matched to therapeutic timelines, and advanced encapsulations that suppress fouling and stabilize performance in vivo.

### 4.2 Miniaturization

The move toward minimally invasive implantation demands ultrathin, flexible, and low‐burden structures. Most current devices have been tested in relatively large tissue spaces, while translation to neural, spinal, or intracranial sites requires geometries that minimize tissue disruption. Reducing device size inevitably decreases effective contact area and output power.^[^ ^31^, ^82^, ^120^, ^145^, ^156^ ^]^ Nanoscale surface texturing and 3D conformal architectures have been proposed to enhance charge density at reduced dimensions, but their long‐term reproducibility and stability remain uncertain. Achieving sufficient energy harvesting while ensuring conformity to complex tissue surfaces remains a major challenge.

### 4.3 System Integration and Artificial Intelligence

In practice, TENGs will not function as stand‐alone devices, but as components within closed‐loop systems combining sensors, power management, and wireless communication. Artificial intelligence could further extend their impact: TENG‐based biosignal acquisition, when paired with machine learning, might enable real‐time pattern recognition of physiological signals, early detection of disorders, and adaptive therapies.^[^ ^42^, ^84^, ^91^, ^119^ ^]^ This requires stable, high‐quality data streams, standardized preprocessing, and robust wireless transmission. Regulatory and ethical issues must also be addressed before such systems can leave the laboratory.

### 4.4 Clinical Translation and Commercialization

Most implantable TENGs remain at the proof‐of‐concept stage. Stable integration with control circuits, power management, and wireless modules has rarely been validated in clinically relevant models.^[^ ^11^, ^106^, ^120^, ^131^, ^138^ ^]^ Regulatory approval requires rigorous documentation of reliability, sterilization, electrical safety, and chronic biocompatibility criteria that existing prototypes seldom meet. Manufacturing also presents obstacles: current devices are typically handcrafted in small batches, limiting reproducibility. Scalable fabrication methods, such as micro/nanofabrication or roll‐to‐roll imprinting, will be essential for reproducible device structures and cost‐effective production.

### 4.5 Outlook

The clinical future of implantable TENGs depends on overcoming three interlinked challenges: i) development of stable or bioresorbable materials with predictable in vivo behavior, ii) miniaturized yet efficient architectures for sensitive anatomical sites, and iii) system‐level integration and scalable manufacturing under regulatory standards. Addressing these issues will determine whether TENGs can move beyond laboratory prototypes and become reliable tools in clinical medicine.

## Conflict of Interest

The authors declare no conflict of interest.

## Data Availability

Data sharing is not applicable to this article as no new data were created or analyzed in this study.
